# ultraLM and miniLM: Locator tools for smart tracking of fluorescent cells in correlative light and electron microscopy

**DOI:** 10.12688/wellcomeopenres.10299.1

**Published:** 2016-12-13

**Authors:** Elisabeth Brama, Christopher J. Peddie, Gary Wilkes, Yan Gu, Lucy M. Collinson, Martin L. Jones

**Affiliations:** 1 Electron Microscopy Science Technology Platform, The Francis Crick Institute, London, UK

**Keywords:** GFP, In-resin fluorescence, Correlative, CLEM, Integrated, Ultramicrotome, Serial block face SEM, ultraLM, miniLM, Smart tracking, Locator tool

## Abstract

In-resin fluorescence (IRF) protocols preserve fluorescent proteins in resin-embedded cells and tissues for correlative light and electron microscopy, aiding interpretation of macromolecular function within the complex cellular landscape. Dual-contrast IRF samples can be imaged in separate fluorescence and electron microscopes, or in dual-modality integrated microscopes for high resolution correlation of fluorophore to organelle. IRF samples also offer a unique opportunity to automate correlative imaging workflows. Here we present two new locator tools for finding and following fluorescent cells in IRF blocks, enabling future automation of correlative imaging. The ultraLM is a fluorescence microscope that integrates with an ultramicrotome, which enables ‘smart collection’ of ultrathin sections containing fluorescent cells or tissues for subsequent transmission electron microscopy or array tomography. The miniLM is a fluorescence microscope that integrates with serial block face scanning electron microscopes, which enables ‘smart tracking’ of fluorescent structures during automated serial electron image acquisition from large cell and tissue volumes.

## Introduction

Correlative light and electron microscopy (CLEM) workflows are now widely used in biomedical research ( Muller-Reichert & Verkade, 2012; Muller-Reichert & Verkade, 2014). CLEM techniques complement fluorescence microscopy (FM) of biomolecular markers in cells and organisms with high-resolution electron microscopy (EM), thus linking molecular function with cell and tissue ultrastructure.

**Figure 1.**
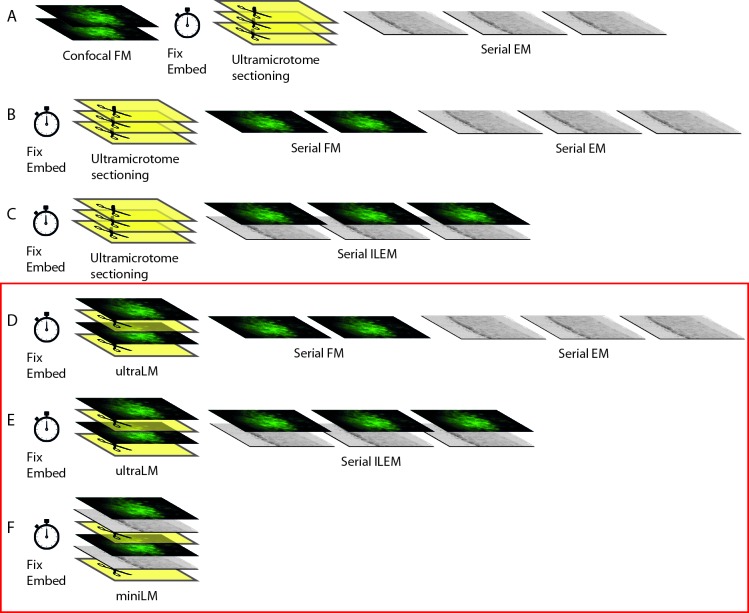
Strategic development of fluorescent cell locator tools for automated correlative workflows. New techniques presented in this manuscript are delineated by a red box. ( **A**) Pre-embedding CLEM workflow uses FM of hydrated cells/tissues, followed by resin-embedding and ultrathin sectioning and EM imaging. ( **B**) The IRF workflow preserves fluorophores, allowing post-embedding FM and EM on the same ultrathin section, thereby improving overlay accuracy. ( **C**) Correlation of fluorescent macromolecules to cellular structure can be further improved and automated by collecting FM and EM images sequentially, and without moving the sample, within an integrated microscope. ( **D**– **E**) Integration of the ultraLM into an ultramicrotome enables ‘smart collection’ of sections containing fluorescent cells/tissues, for subsequent imaging in separate ( **D**) or integrated ( **E**) light and electron microscopes. Automated ‘smart tracking’ of fluorescent cells/tissues is achieved by integration of the miniLM into a SBF SEM ( **F**).

To image a biological sample in the electron microscope, it must first be protected from the vacuum in the EM chamber, usually by embedding in resin or ice. The first CLEM workflows linked pre-embedding FM of samples with post-embedding imaging in the EM ( Figure 1A) ( Polishchuk *et al.*, 2000). Recent CLEM workflows preserve the fluorophores during resin-embedding, enabling post-embedding FM and EM on the same sample ( Bell *et al.*, 2013; Johnson *et al.*, 2015; Kukulski *et al.*, 2011; Nixon *et al.*, 2009; Peddie *et al.*, 2014a; Watanabe *et al.*, 2011). These in-resin fluorescence (IRF) blocks can be cut using an ultramicrotome, to produce ultrathin IRF sections of 50–200 nm, which can be imaged sequentially with stand-alone fluorescence and electron microscopes ( Figure 1B), or *in situ* in an integrated light and electron microscope ( Figure 1C) ( Peddie *et al.*, 2014b). Our previous work demonstrated that imaging IRF sections with an integrated light and scanning electron microscope (ILSEM) results in high precision correlation of fluorescent protein (FP) signal to cell organelles ( Peddie *et al.*, 2014a).

To take full advantage of IRF samples containing both fluorescence and electron signals, several steps in the CLEM workflow must be further developed. The first key step is ultramicrotomy, which requires integration of an FM module to detect fluorescent cells at the blockface during sectioning, thus enabling section collection from specific regions of interest (ROIs) ( Figure 1D,E). The second key step is imaging. Though FM modules have been integrated into both transmission EMs (TEMs; Agronskaia *et al.*, 2008) and scanning EMs (SEMs; Liv *et al.*, 2013), to date there has been no such integration into volume EMs. These automated 3D EMs are capable of collecting thousands of images through large sample volumes, using either an ultramicrotome (serial blockface SEM; SBF SEM) or an ion beam (focused ion beam SEM; FIB SEM) to slice through the sample within the SEM chamber. However, the complex chamber geometry limits the possibilities for FM integration as very little space remains after the electron column, sample stage, electron detectors, gas injection systems, and ion column (FIB SEM) or ultramicrotome (SBF SEM) have been taken into account.

In response to this challenge, we designed two miniaturised light microscopes, designed to fit into tight spaces and integrate into CLEM workflows. The ultraLM™ integrates with an ultramicrotome to locate fluorescent cells during section preparation ( Figure 1D,E), and the miniLM™ integrates with the SBF SEM ( Figure 1F) to locate cells during volume EM acquisition. These microscopes will enable future automation of 3D correlative data collection via fluorescence-guided ‘smart’ image acquisition and object tracking.

## Results

### Design requirements for the ultraLM

The ultraLM design took into account the following design principles: the microscope must be positioned in front of the resin for imaging; at a safe distance above the diamond knife to avoid damage; without obscuring the operator view of the sections in the boat; whilst allowing access to the boat for section retrieval; with a resolution of at least 1 µm for subcellular feature recognition, and a field of view of at least 500 × 500 μm, to view most or all of the resin blockface; without adding vibration into the system.

### Build and specification of the ultraLM

**Figure 2.**
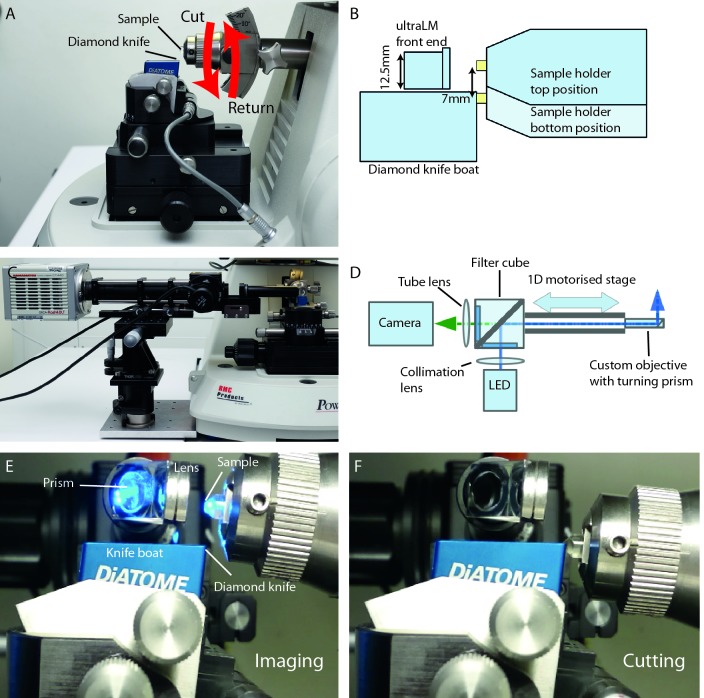
Build and integration of the ultraLM into an ultramicrotome. ( **A**) Side view of an ultramicrotome showing the relative positions of the sample and diamond knife. The motion of the sample is indicated with red arrows. Each time the sample goes through the ‘cut’ motion, an ultrathin section is removed from the blockface and floats onto water. ( **B**) Schematic of the FM imaging position showing the space limitations around the knife and sample, and the position of the ultraLM customised lens. ( **C**) Front view of the ultraLM microscope showing the mounting of the apparatus onto a custom plate bolted to the base of the microtome. ( **D**) Schematic highlighting the main components and the optical path of the ultraLM. ( **E**– **F**) Close-up side view of the ultraLM during operation, showing the imaging ( **E**) and cutting positions ( **F**).

In the ultramicrotome, the resin block is secured in a horizontal sample arm. During the cutting stroke, the resin block is advanced toward the diamond knife by a predetermined distance (section thickness; typically 50 to 500 nm), and the motion of the cutting cycle moves the resin block down past the diamond knife to cut a single section. On the return stroke, the block is temporarily retracted by a set distance to prevent contact with the diamond knife, and returned to the top of the arc to begin the next cutting cycle ( Figure 2A). The space constraints imposed by the geometry of the microtome and the diamond knife ruled out the use of a bulk objective for the ultraLM ( Figure 2B). Instead, a custom imaging lens was designed and constructed using a small diameter achromatic lens, mounted to achieve a diameter of 12.5 mm. A 90° turn of the imaging path allowed the microscope body to be mounted to the side of the microtome ( Figure 2C,D). Since a larger lens was preferred for better image quality, the ultraLM was positioned at the top of the cutting arc, as far away from the diamond knife as possible ( Figure 2E,F). It is important to note that this lens configuration obstructs the view of sections at the knife edge. Rather than compromise image quality, motorisation of this axis meant that an automatic lens retraction mechanism could be built into the microscope, allowing the user a clear view of the sections when required ( Figure 2C).

A small custom microscope was assembled using Thorlabs 30mm cage components, with a GFP filter set, a 490 nm excitation LED and an sCMOS camera. The ultraLM was specified with a f=150 mm tube lens to give an overall system magnification of 18.75 and a field of view (FOV) of ∼710 μm. The numerical aperture (NA) of the lens was 0.5, giving a theoretical resolution of ∼600 nm for GFP excitation at 488 nm. The lens was characterised using fluorescent beads dried on a coverslip, giving a measured magnification of 18.66 (n=3) and a FOV of 713.4 µm, in good agreement with the theoretical values. The lateral resolution was measured using three different methods: The distance between pairs of resolvable beads; calculation from the point spread function (PSF) of beads imaged by ultraLM (using 200 nm and 500 nm beads); and the de-convolved PSF image of 1 µm beads. All three showed a lateral resolution for the ultraLM of approximately 1.8 µm. The depth of focus was established as ±15 µm using 1 µm beads, where the depth of focus was defined as the range over which the signal retained ⅔ of the intensity and contrast when compared to the focal plane.

For automated ultraLM image acquisition, the microtome driver board was modified to output a signal when the sample arm reached the top of the cutting stroke, which was connected to the camera’s external trigger input. The sample arm was set to move very slowly through this imaging position, to allow image acquisition without disturbing the mechanical stability of the sample arm movement. One image was captured per cutting cycle, and the images were displayed on the microtome control PC and automatically saved using customised software (Python and PyQt) built on the Micromanager core ( Edelstein *et al.*, 2014) in order to interact with the camera.

### Proof of principle application of the ultraLM

**Figure 3.**
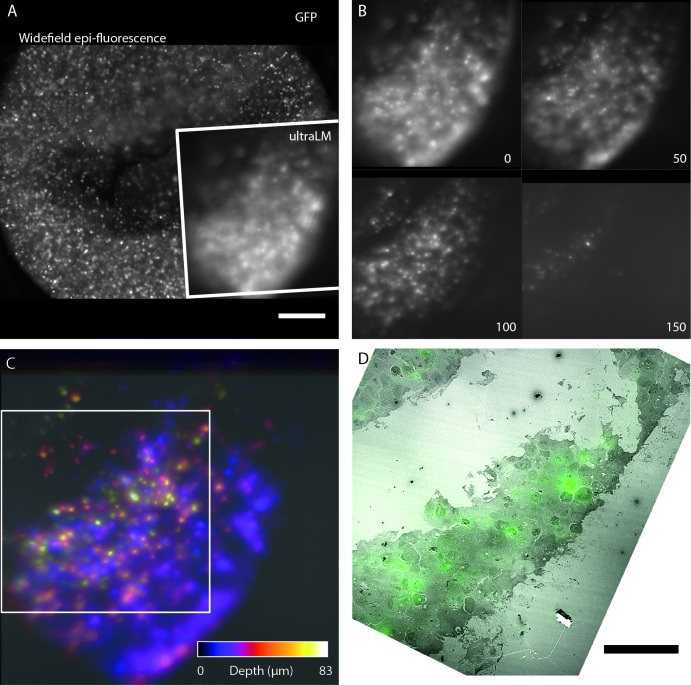
Fluorescence imaging during ultramicrotomy with the ultraLM. Proof-of-principle ultraLM operation was demonstrated using IRF blocks containing HeLa cells expressing GFP-H2B. ( **A**) Low magnification widefield epi-fluorescence image of the blockface before sectioning, with inset showing the region imaged by the ultraLM. Scale bar 200 µm. ( **B**) Blockface images acquired with the ultraLM, from a serial imaging and cutting run. Every 50 ^th^ image is shown, at a Z separation of 25 µm. Sectioning was performed at 500 nm thickness with a diamond knife. ( **C**) Depth-coded maximum intensity projection of image stack shown in panel **B**, indicating positions of cells within the resin. White box delineates region of overlay of the final blockface image of GFP-H2B expressing cells (green) onto the last section cut from the block and imaged in a TEM ( **D**), showing that the ultraLM image can be used to locate fluorescent cells during sectioning. Scale bar 100 µm.

Cells expressing GFP-H2B were embedded using our IRF protocol ( Peddie *et al.*, 2014b) ( Figure 3). The blocks were trimmed and mounted in the sample holder arm, and the ultraLM was inserted and positioned using the xyz stage. A small step was cut into the blockface using a glass knife to aid focusing of the ultraLM at the resin surface. After swapping the glass knife for a diamond knife, the ultramicrotome was set to cut sections at a thickness of 500 nm, and a serial sectioning and imaging run was initiated ( Figure 3B,C and Movie S1). As sectioning progressed, GFP-H2B positive nuclei deeper within the sample appeared initially as blurred objects that came into focus as they approached the focal plane at the block surface, and then disappeared as they were removed in sections ( Figure 3B). A depth-coded maximum intensity projection showed the positions of the cells through the depth of the sectioned volume ( Figure 3C). After the final FM image was acquired, the next section was collected and imaged in the TEM. The same cells were located in both FM and TEM images, and the images were overlaid to demonstrate how the technique could be used to locate sparse fluorescent objects within a cell pellet or within a larger tissue sample for subsequent EM analysis ( Figure 3D).

### Design requirements for the miniLM

The miniLM design took into account the following design principles: the microscope must be positioned directly above the resin block for imaging; at a safe distance from the backscattered electron (BSE) detector, sample and diamond knife to avoid damage; without obscuring the view of the block during electron imaging; and without adding vibration into the system. The miniLM must integrate into the cutting and imaging cycle of the SBF SEM; must be conductive, non-magnetic and vacuum-compatible; with a resolution of at least 1 µm for subcellular feature recognition, and a field of view of at least 500 × 500 μm, to view most or all of the resin blockface. The bulk of the microscope (illumination and detection systems) must be positioned outside the vacuum chamber due to space constraints, requiring a mechanism for feeding light into and images out of the vacuum chamber. The system should also involve minimal adaptation of existing components in the electron microscope to allow it to be retro-fitted into existing SBF SEMs.

### Build and specification of the miniLM lens

**Figure 4.**
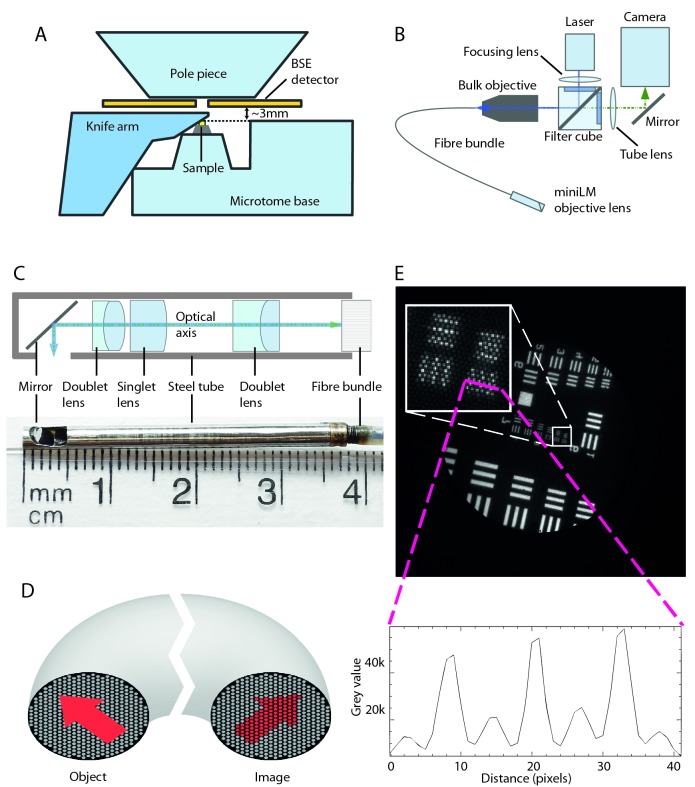
Miniaturisation of FM to create the miniLM for SBF SEM integration. ( **A**) Schematic of the interior of the 3View SBF SEM chamber showing relative positions of major components and the location of the 3 mm gap between the top of the sample and the bottom of the BSE detector. ( **B**) Schematic of the optical arrangement of the miniLM. ( **C**) Schematic of the custom lens in the miniLM alongside a photograph showing the physical dimensions. The miniature microscope objective is attached to the end of a coherent fibre bundle, which serves to feed the image out of the vacuum chamber. ( **D**) Schematic of the coherent fibre bundle. An image presented to the proximal end of the fibre is relayed in a spatially coherent manner to the distal end. Note the resultant pixellation of the image due to the finite number of fibre cores. ( **E**) Transmission image of a USAF target to demonstrate the resolution of the miniLM. The smallest lines on the target are 2.18 µm wide with equal separation distance, which we were able to resolve. The inset shows a line profile taken through the lines of Group 7, Element 6 of the USAF resolution target, confirming a resolution of better than 2.18 µm. The smaller background peaks were caused by the pixellation due to the discrete fibre cores. The target was illuminated with a white light LED through an objective of similar NA to the miniature objective.

The miniLM had more stringent size requirements than the ultraLM due to the geometry and spatial restrictions of the SBF SEM chamber ( Figure 4A). The design of the miniLM was inspired by fibre-coupled endoscopy imaging systems, which are also designed to fit into tight spaces ( Coda *et al.*, 2015; Engelbrecht *et al.*, 2010; Flusberg *et al.*, 2005). Our solution consisted of a custom-built miniature objective attached to a coherent fibre bundle, which fed through the vacuum chamber door to a custom-built epi-fluorescence microscope ( Figure 4B).

The miniaturised objective lens was designed to fit into the gap between the post-column BSE detector and the block surface, calculated to be ∼3 mm. Due to space restrictions, the optical path had to undergo a 90° turn in close proximity to the imaging plane, which presented an additional challenge for lens miniaturisation. The objective lens was mounted in a 2.8 mm diameter hollow non-magnetic stainless steel ferrule of 36 mm length and consisted of two doublet lenses and one singlet lens ( Figure 4C), designed and manufactured externally by Kingsview Optical Ltd (Rye, UK). The ferrule attached to the fibre bundle with a thread and lock-ring mechanism. This semi-permanent attachment was chosen to allow adjustment of the image plane of the miniature objective with respect to the fibre end. All lens surfaces were coated with a broad band (480 to 610nm) multi-layer coating. The 90° turn was achieved using a silver mirror positioned 2 mm from the end of the ferrule ( Figure 4C). An elliptical hole in the side of the tube allowed imaging to take place with a focal plane 0.1 mm outside the ferrule. Due to the extremely small ferrule diameter, the lens achieved an object side NA of 0.21 (approximate resolution at 490 nm, d = 1.42 µm). The FOV was specified to be 600 µm and the magnification of the lens as 2.05 to match the image to the diameter of the fibre bundle (1.10 ± 0.08 mm).

The coherent fibre bundle (also known as an imaging fibre bundle) was constructed such that the relative positions of all the fused fibres were maintained throughout its length, faithfully relaying a pattern of illumination from one end to the other ( Figure 4D) ( Hopkins & Kapany, 1954). The finite size of the individual fibre cores (approximately 3.2 µm diameter), coupled with the magnification of the miniature objective, imposed a second limit on the resolution of the complete system. For the given values, this was calculated as ∼1.7 µm, closely matching the resolution of the lens.

The actual lateral resolution of the lens assembly was tested using a USAF target ( Figure 4E). The thinnest lines of the target could be resolved, indicating a resolution better than 2.18 µm. The actual magnification of the objective was measured as 2.07, with a FOV of 515 µm. The depth of focus was determined using the same target but with the axial position of the microscope motorised. Stepping the motor at 3 µm intervals, the target was in focus over a depth of 39 µm. However, theoretical calculations suggested an axial resolution of ∼11 µm for a lens with an NA of 0.21. This discrepancy could be explained because the finite size of the fibre cores was larger than the focal spot size at any point in the image. Therefore, a single fibre core could not discriminate between focal plane positions any better than the lens itself.

### Integration of the miniLM into the SBF SEM

**Figure 5.**
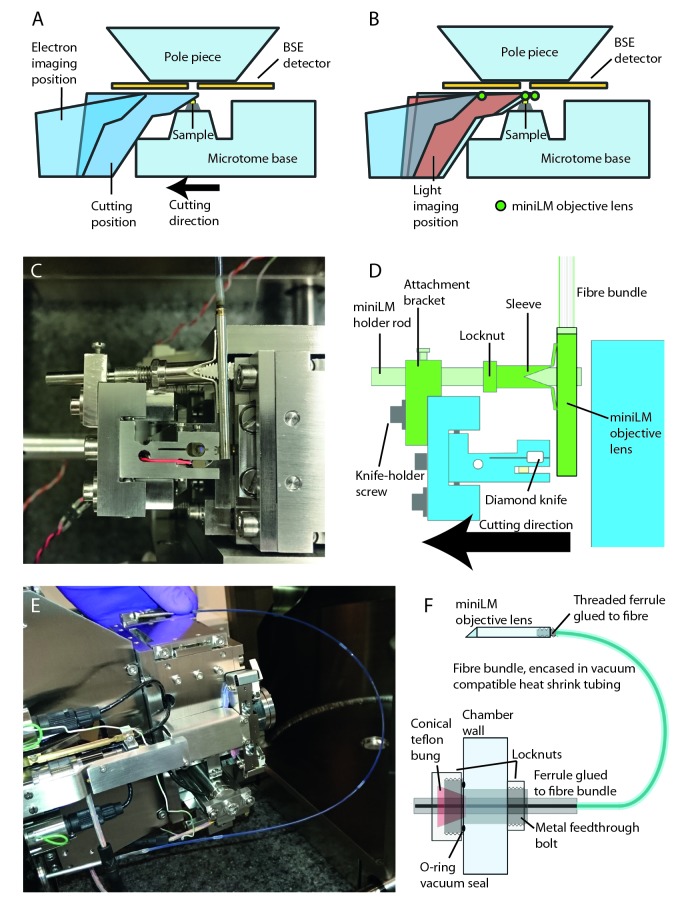
Physical integration of the miniLM with the SBF SEM. ( **A**) Schematic of the interior of the SBF SEM showing the knife arm in the cutting position and the electron imaging position. ( **B**) Addition of an intermediate position at which the motion is paused to enable FM imaging with the miniLM, which is denoted by a green circle, as viewed in cross-section. ( **C**) Photograph of the SBF SEM knife-holder region viewed from above, described further in schematic ( **D**), indicating the SBF SEM microtome parts (in blue) and the custom miniLM parts (in green). ( **E**) Photograph showing the custom vacuum feedthrough and fibre bundle *in-situ*, described further in schematic ( **F**). The airtight vacuum feedthrough is based around a modified Swagelok style compression fitting inserted into an existing bolt hole in the SBF SEM door.

The SBF SEM vacuum chamber contains an ultramicrotome that holds the resin-embedded sample just below the electron column and BSE detector for electron imaging ( Figure 5A). The diamond knife, mounted in a holder, passes over the imaged surface to cut ultrathin sections from the blockface. The knife motion is controlled by a lever arm that is driven by a cam for fast movement outside the cutting window, and a piezo motor for slow precise movement within the cutting window. The movement of the knife-holder across the block surface, and the space limitations imposed by the BSE detector, prevented permanent mounting of the miniLM directly above the sample. For this reason, the miniLM was designed to piggyback on the knife-holder ( Figure 5B), thereby taking advantage of the existing ultramicrotome motors for reproducible positioning of the miniLM above the block ( Figure 5B). For simplicity, the miniLM was attached using an assembly designed to slide onto one of the fixing screws attaching the knife-holder to the lever arm ( Figure 5C,D). The height of the knife was fixed, while the sample moved upwards by the selected section thickness for each cutting cycle. The blockface position was thus fixed in space and the focus needed only to be set when the miniLM was installed. This could be done at atmospheric pressure with the chamber door open, after alignment of the sample for cutting.

The image was relayed out of the SEM chamber through the coherent fibre bundle, fed through the chamber door using a custom vacuum-tight fitting ( Figure 5E,F) ( Abraham & Cornell, 1998). Due to the limited space available within the chamber, the fibre was bent into a semicircle with a radius close to the minimum permissible bend radius. The external fibre end was imaged with an sCMOS camera attached to a custom built epi-fluorescence microscope assembled using the Thorlabs cage system. The magnification of the combined bulk and miniLM objective was calculated to be 17.28.

**Figure 6.**
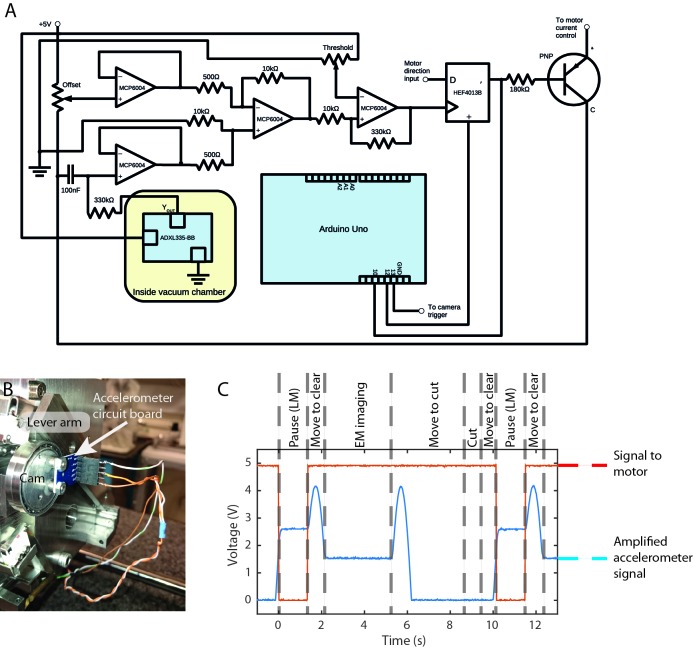
Electronic integration of the miniLM with the SBF SEM. ( **A**) Circuit diagram of the electronic circuit and Arduino interface used to integrate the miniLM into the SBF SEM. ( **B**) Accelerometer chip mounted on the microtome cam. ( **C**) Signal behaviour during the cutting and imaging cycle for the accelerometer and motor current. Note that the accelerometer signal has been amplified for increased sensitivity in the region of motion of interest, causing saturation and clipping at 0 V for part of the cycle.

To automate miniLM imaging within the SBF SEM imaging workflow, an external control circuit was built and minor modifications were made to the microtome electronics ( Figure 6A). This enabled us to interrupt the power supply to the motor driver as the miniLM passed across the blockface. The exact ‘stop’ position was determined using an accelerometer attached to the cam driving the lever arm ( Figure 6B) and visual feedback from the camera. The outcome was a cutting cycle consisting of: cut section; move to LM imaging position; acquire LM image; move to cleared knife position; acquire EM image; return to near position. As before, the LM image was captured and displayed on a control PC, and automatically saved using customised software (Python and PyQt) and the Micromanager core to interact with the camera. Figure 6C shows the amplified accelerometer signal, and the signal sent to the motor control as the microtome cycled through this sequence. Note that the signal was amplified such that the lower part was saturated and clipped at 0 V. The inclusion of the Arduino allowed programmatic control of the time window during which the LM image was acquired.

### Proof of principle application of the miniLM

**Figure 7.**
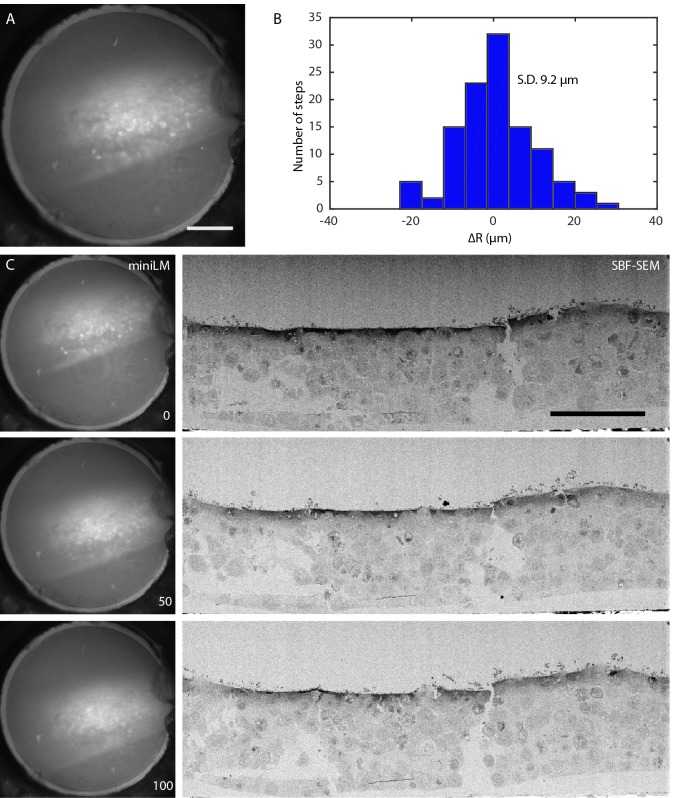
Automated *in vacuo* serial LM-EM imaging using the miniLM. ( **A**) Low magnification raw image of the distal end of the fibre bundle, showing that the miniLM resolves individual GFP-H2B expressing HeLa cells. Scale bar 100 µm. ( **B**) Histogram showing the distribution of the inter-step deviation of the miniLM acquisition position, with a standard deviation of 9.2 µm. ( **C**) Sequence of raw miniLM images and matching electron images of the cell layer, showing every 50 ^th^ image from an automated serial LM-EM imaging and cutting run, demonstrating that the miniLM can detect fluorescent cells *in vacuo* during an SBF SEM data acquisition. Scale bar 100 µm.

IRF blocks containing cells expressing GFP-H2B were also used for proof-of-principle work with the miniLM ( Figure 7). The blocks were mounted, clamped into the SBF SEM sample holder, and aligned to the diamond knife. The focal plane of the miniLM was optimised by adjusting the height of the lens assembly above the block, and the miniLM was clamped into place ( Figure 7A). The door was closed and the microscope pumped to a vacuum pressure of 5 Pa. The electron imaging conditions were set as described in the methods section, and a serial LM/EM imaging run performed at 100 nm slice thickness. The positioning accuracy of the miniLM was recorded over 150 cutting cycles, and was found to vary slice to slice with a standard deviation of 9.2 µm ( Figure 7B and Movie S2). This error could be attributed to drift caused by mechanical instability in the mounting and jitter caused by electronic noise from the circuit. Improved mechanical mounting and shielding of the circuit and transmission lines from environmental electromagnetic noise could reduce these effects in future designs.

A sequence of 100 alternate fluorescence and electron images was collected ( Figure 7C and Movie S3). The miniLM did not affect the cutting or electron imaging functions of the SBF SEM, and did not introduce any noticeable vibration into the system.

## Discussion

As the availability of dual-contrast samples containing both fluorescent markers and electron contrast becomes more widespread, the correlative imaging field is rapidly shifting in a new direction. In response, we present two novel fluorescence-guided cell locator tools, in the form of miniaturised fluorescence light microscopes that complete correlative imaging workflows. The first is for use with an ultramicrotome, to enable fluorescent cell localisation during ultrathin sectioning. The second is for use with an SBF SEM, enabling ‘smart tracking’ of fluorescent cells during automated serial imaging runs. Both the ultraLM and the miniLM were conceived and designed with the intention of retrofitting into existing systems. As such, our approach should enable the correlative imaging community to replicate these designs and integrate them into their own platforms.

A proof-of-principle serial imaging and cutting run for both the ultraLM and miniLM is presented, using a pellet of FP-expressing cells embedded in resin blocks. In these IRF samples, most cells express the fluorophore, enabling us to demonstrate the concepts of automated sequential LM imaging using the ultraLM, and sequential LM-EM imaging using the miniLM. However, for most applications, we would expect the IRF blocks to contain only a small proportion of fluorophore-expressing cells. This is often the case when cultured cells have been transfected with a fluorescent genetic construct, infected with a fluorescent infectious agent, or when using a genetically-modified model organism with a sparse subset of FP-labelled structures such as blood vessels or neurons.

In sparsely-labelled cell populations, the ultraLM allows the operator to identify areas of interest and collect sections only from regions containing fluorescent signal, without repeatedly interrupting sectioning to screen individual sections using a separate fluorescence microscope. Since only sections containing cells of interest are selected for subsequent electron imaging, the downstream imaging steps are also expedited. The ultraLM forms a basis for several design iterations which will be pursued in future work: integration with a cryo-ultramicrotome for targeted collection of cryo-sections containing fluorescent cells for Tokuyasu immunolabelling or cryo-electron tomography; integration with automated ultramicrotomes for smart tracking of fluorescent cells for correlative array tomography ( Hayworth *et al.*, 2014; Schalek *et al.*, 2011); and potentially as a stand-alone serial fluorescence imaging platform for 3D FM through volumes, similar in concept to high resolution episcopic microscopy (HREM) ( Henkelman *et al.*, 2016; Weninger & Mohun, 2007).

**Figure 8.**
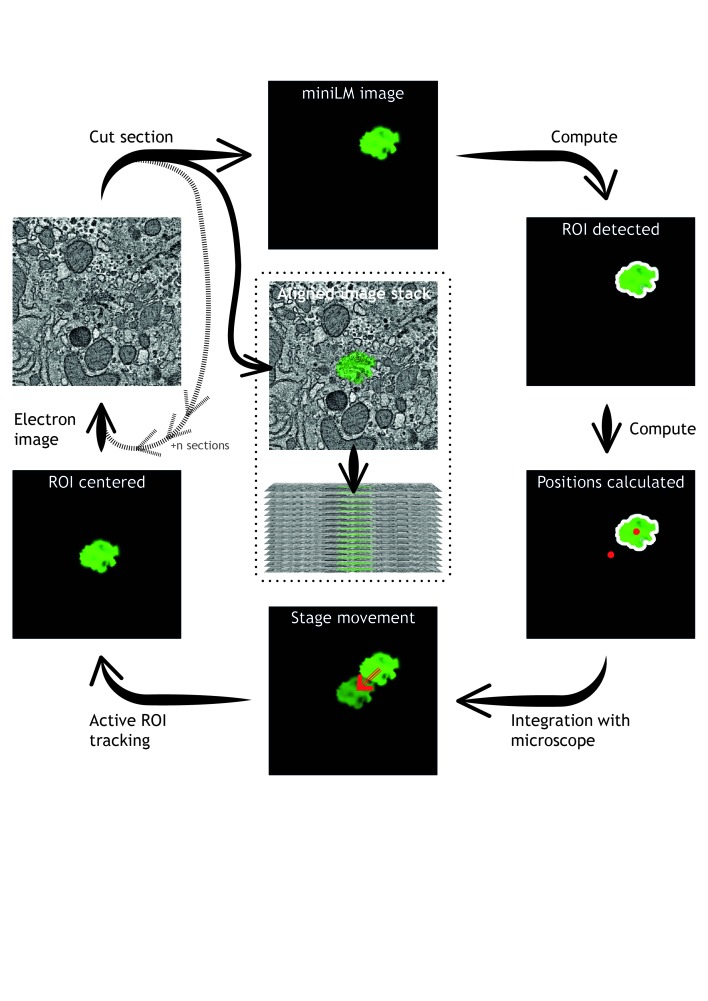
Automation of integrated 3D light and electron microscopy using the miniLM. Advances in automated algorithms that detect fluorescent cells in miniLM images will enable smart tracking of regions of interest during an SBF SEM data acquisition.

In sparsely-labelled cell populations, the miniLM enables smart-tracking of fluorescent cells and structures *in vacuo* during a SBF SEM imaging run. The miniLM can be used manually to check the position of sparse fluorescent regions at the blockface, followed by manual adjustment of specimen position to track these regions through the volume, without breaking vacuum and removing the block for imaging on a separate fluorescence microscope. Future iterations of the miniLM will incorporate novel algorithms for automatic detection of fluorescent signal at the block surface, extracting the positions of ROIs and using them to drive the specimen position to track sparse fluorescent structures through the volume automatically ( Figure 8). The miniLM design is also compatible with future integration into FIB SEM and cryo-FIB SEM platforms.

Though we have demonstrated successful optical, mechanical, and electronic integration into existing instruments, there are some limitations that may be overcome in the next generation of miniaturised LMs. Improvements in numerical aperture and optical performance may be possible with advances in miniaturisation of lenses and multi-core imaging fibres. Alternative methods for delivery of the excitation light to the miniLM would avoid the large losses associated with fibre-coupling, and could enable use of LEDs rather than lasers. Though a multi-component assembly was used for the miniLM holder during development, a single-piece holder would improve stability over long-term imaging runs. Finally, though we discounted free-space coupling of the fluorescence images out of the vacuum chamber due to a lack of vacuum chamber ports in line-of-sight, future design iterations would ideally remove the need for a coherent fibre bundle, thereby lifting the resolution limitation imposed by the individual fibre cores. This would enable acquisition of matching 3D LM and 3D EM image stacks with a voxel resolution that would allow direct correlation of FPs to subcellular organelles through large tissue volumes. The ability to perform this in an automated and integrated way would address one of the grand challenges of biological correlative imaging experiments.

## Methods

### Preparation of 3D test sample: Fluorescent cells embedded in resin

HeLa cells expressing GFP-H2B were prepared using an IRF protocol as previously described ( Peddie *et al.*, 2014a; Peddie *et al.*, 2014b).

### ultraLM imaging system

A custom imaging lens was designed and constructed using a small diameter achromatic lens (Thorlabs, A240-A) mounted in two lens adapters (Thorlabs, LMRA10). This assembly was glued directly onto an anti-reflection coated right-angle prism (Thorlabs, PS914L-A), which was in turn glued onto a 0.5 inch lens tube. The lens tube of the objective lens was mounted on a 1D translation stage (Thorlabs, CT1) and motorised using a DC servo motor (Thorlabs, Z825B). The rest of the microscope consisted of a GFP filter set (Chroma, 49002), an excitation LED (Thorlabs, M490L2), an f=150 mm tube lens, and an sCMOS camera (Hamamatsu Flash 4.0 V2). The cage-assembled microscope was mounted on an optical dovetail rail (Thorlabs, RLA150/M) with two dovetail rail carriers (Thorlabs, RC1) attached to two cage plates. The rail was mounted on two stages, one for the focus direction (Thorlabs, PT1/M, 25 mm travel), and one for vertical adjustment (Thorlabs, MVS005/M, 13 mm travel). The stages were screwed on an adjustable height platform on a 1.5 inch post (Thorlabs, C1519/M, P50/M, and PB4/M). The post was clamped down onto a custom plate manufactured in-house, which was firmly attached to the base of the microtome. The clamp mounting and the adjustable height platform also allowed for coarse microscope positioning.

To synchronise image acquisition at the top of the cutting arm cycle, a trigger signal was taken from the microtome driver board, and passed to the camera external input. The ultramicrotome EPROM chips were reprogrammed by the manufacturer (RMC Boeckeler, Tucson AZ) to control the behaviour of the microtome arm during cutting and imaging cycles.

The ultraLM images were acquired using a custom Python based GUI with a Qt designed front end calling Micromanager to interface with the camera. For data acquisition, the imaging was synchronised via the external camera trigger using a signal from the microtome.

### ultraLM proof-of-principle serial imaging run

To accurately focus on the surface, the resin block was trimmed using a glass knife that was positioned off-centre to trim from only one half the block, thereby creating a stepped surface. The glass knife was then exchanged for a diamond knife, and aligned to the block in preparation for sectioning. The step on the block surface was used to mechanically focus the utraLM using the upper microtome light for illumination. With the microscope aligned and focused, the cutting cycle was started, at a slice thickness of 500 nm (large enough to demonstrate a change in fluorescent signal in the image between each section), and the custom imaging software set to acquire a fluorescence image after receiving the trigger signal from the microtome at the top of the cutting stroke.

### miniLM imaging system

The miniLM lens was designed and manufactured by Kingsview Optical Ltd (Rye, UK). The miniLM ferrule was clamped into a rod, which in turn was fixed to a custom-designed bracket. This was mounted on the back of the 3View knife holder, at a small angle to the horizontal, allowing some vertical (focal) adjustment of the microscope.

A 50,000 core coherent fibre bundle (Fujikura FIGH-50-1100N) was attached to the back of the lens assembly. To protect the fibre from external damage along its length, it was encased in vacuum-compatible FEP heat shrink (LewVac, A-FEPT-1.39). To avoid contamination and damage at the feedthrough end of the fibre, it was glued into a long metal ferrule with the fibre end slightly recessed, which was then fed through a custom Swagelok assembly, designed to fit through a spare port in the chamber door. To protect the fibre end facets during the assembly process, special cleaning glue (First Contact) was applied and then removed post-assembly.

The image created on the bare fibre end outside of the vacuum chamber was imaged using a custom-built microscope consisting of a 10x air objective (Olympus UPLFLN 10x), a fluorescence filter set (Thorlabs MF469-35, MD498, MF535-22), an f=150 mm tube lens and an sCMOS camera (Hamamatsu Flash 4.0 V2). The sample was illuminated via the filter cube, bulk objective, fibre bundle and miniature objective using the expanded beam of a 488 nm laser (Coherent, Obis 488 LX, 50 mW). The microscope was assembled using Thorlabs 30 mm cage components. Initial tests indicated autofluorescence in the fibre bundle when illuminating, with a peak at 510 nm, so the emission filter was selected to remove the majority of this autofluorescence.

The miniLM images were acquired using a custom Python based GUI calling Micromanager to interface with the camera with a Qt designed front end. For data acquisition, the imaging was synchronised via the external camera trigger using a signal from the Arduino.

### miniLM lens characterisation

The pixelation inherent in the use of the fibre bundle, coupled with the low light efficiency of the system, ruled out the use of fluorescent beads for characterising the lens. In addition, the short working distance prohibited the use of a fluorescent target, so an alternative method was used to give an accurate measure of lens performance. A USAF target was set up in transmission and illuminated with an LED through an objective with a similar NA to the miniLM objective (0.2). The bulk objective used in the custom microscope to image the fibre end had a magnification of 8.33 because the tube lens was 30 mm shorter than the Olympus standard. Measuring a line profile across the image and using the camera specifications (pixel size 6.5 µm, square chip of 2048 pixels), the size of the fibre was calculated to be 1067 µm. The lines inside the image (group 4, element 2) were 27.84 µm in width. After applying smoothing filters to blur the fibre cores in the image, plotting a line profile across the lines showed them to be 481 µm wide on the chip. The total magnification of the system (combined bulk and miniature objective) was therefore 17.28. Factoring out the bulk objective’s magnification resulted in a miniature objective magnification of 2.07. The actual field of view of the system was therefore 515 µm in diameter.

To determine the depth of field of the assembly, the focus direction of the stage for positioning the microscope in front of the target was motorised using a DC motor (Thorlabs, Z825B). Using the software control of the motor, the microscope was stepped in 3 µm increments to bring the target in and out of focus, and an image taken at every position. The images were imported into Fiji and a rectangular line profile was plotted across the smallest lines of the target. For practical application of the microscope, in tracking fluorescent objects through the resin volume, the depth of focus was defined as the distance over which the user perceived the cells as focused and well-separated objects. Due to the nature of the fibre bundle, the line profiles over the unprocessed image displayed a regular structure of peaks, representing the individual fibre cores. For a more accurate estimate of focus, we defined the image to be out-of-focus when the peaks were no longer distinct from this overlaid structure. This definition coincides well with what a user perceives as in focus.

### miniLM drive electronics

To acquire information about the position of the 3View knife arm, an accelerometer chip was used (Analog Devices, ADXL335-BB). Since the angular range of the lever arm was relatively small, the accuracy of the angular measurement would not have been sufficient to position the microscope accurately for miniLM image acquisition at the blockface. The arm was, however, driven by a cam, which moved through a much larger range of angles during the cutting process, so the chip was attached to this instead. Two spare wires in the existing auxiliary cable feedthrough on the door were used to provide a 5V supply to the chip and to readout the voltage from the accelerometer. Ground was provided via a cable connected to the chamber door.

The accelerometer chip provided an analog signal of approximately 330 mV/g, which gave a signal range of approximately 500 mV over the angular movement range of the cam. To further increase the sensitivity of the readout, we subtracted the offset (minimum voltage output) and amplified the signal. An instrumentation amplifier based on the MCP6004 chip subtracts a fixed voltage from the accelerometer readout to remove the offset. The offset could be adjusted via a potentiometer and was set to approximately the minimum value detected by the accelerometer during operation. The output of the operational amplifier behaved according to V _OUT_ = (V _IN2_ - V _IN1_) * R _1_/R _2_ + V _REF_. V _REF_ is connected to ground.

To set the stopping position for miniLM imaging at the blockface, we used a Schmitt trigger circuit based on the remaining op amp on the MCP6004 chip. This compared the threshold set by a second potentiometer (adjusted using the visual feedback of the light microscope for the desired stopping position) to the amplified accelerometer signal, being LOW below threshold, and HIGH otherwise, as well as introducing approximately 150 mV of hysteresis, thus helping to avoid any double-triggering caused by noise.

The mounting position of the accelerometer was optimised such that the required threshold voltage for stopping was safely on the slope of increasing voltage. As a result, the voltage output described a curve that increased and decreased again during the cutting stroke, with a total range of approximately 250 mV. We therefore chose R1 and R2 to be 10 kΩ and 50 kΩ respectively, to achieve an amplification of 20 to exploit the full 5 V range of the circuit.

To interface with the microtome electronics, we made electrical connections to a chip on the microtome controller board of the 3View microtome, one connection to obtain information about the direction of motion of the lever arm, and a second to cut the motor current externally to achieve the additional stopping position for miniLM imaging to take place.

In order to avoid the knife arm stopping again on the downward slope, we used a D-type flip flop (HEF4013B). The motor direction input was connected to the data (D) input, while the comparator output provided the clock pulse (CP, only triggers on a rising edge), resulting in the motor current being cut when the threshold value for the accelerometer was reached, providing that the directional input of the motor was LOW. After the miniLM image was acquired, the clock pulse was fed into an Arduino Uno microcontroller board, which in turn sent a pulse to the set direct input (SD) of the flip flop to restart the motor after a specified delay. This allowed the user to set an adequate stoppage time for miniLM imaging to take place via the Arduino software. We also added a low pass filter with a cut off frequency of 4.8 Hz to filter the accelerometer signal. The circuit was powered using an external 5 V power supply.

### Physical alignment of the miniLM prior to an integrated imaging run

The alignment of the miniLM in the SBF SEM was carried out in two phases. Firstly, the height of the miniLM lens was adjusted relative to the sample. To do this, the lever arm was manually moved to place the knife-holder in the light imaging position and fixed in place. Then, the lens holder was adjusted until the sample was in focus. In a second step, the stopping position during the cutting stroke was set. After moving the sample down by a small amount to avoid cutting the sample or damaging the knife during the adjustment, the microtome was set to start moving across the blockface and the potentiometer of the electronics circuit was adjusted until the fluorescence signal was central in the field of view of the images taken at the stopping position. With the adjustments finished, the door of the SEM chamber was closed and the chamber pumped to 5 Pa partial pressure of nitrogen gas. The external epi-fluorescence microscope, mounted on a vertical breadboard, was positioned in front of the chamber door and the bulk objective was aligned to the fibre end.

### miniLM serial imaging run

The integrated light and 3D EM image stack was collected using the miniLM attached to a 3View2XP (Gatan, Abingdon) microtome in a Sigma VP SEM (Zeiss, Cambridge). The trimmed IRF block was attached to a specimen pin using conductive epoxy resin (Chemtronics CircuitWorks CW2400), with the cell layer aligned perpendicular to the direction of cutting. The laser power at the sample level was set to ∼2.5 mW and the exposure time set to 500 ms. The delay time until the motor was restarted at the miniLM imaging position was set to 1.5 s. The serial imaging run was set up and started using the 3View microtome control software Digital Micrograph (version 2.3, Gatan Inc.), and the miniLM control software was then started. BSE images were acquired at a resolution of 1024 × 2048 pixels (horizontal frame width of 257.14 µm; pixel size of 250 nm) using a 10 µs per pixel dwell time and 200 nm slice thickness. The SEM was operated at a chamber pressure of 5 Pa, with high current mode active, at an indicated magnification of 500. The 120 µm aperture was used, at an accelerating voltage of 1.4 kV. Fluorescence and electron images were collected sequentially from a total of 500 slices, representing an overall depth of 10 µm and total volume of 1,322,445 µm ^3^. The cell layer, nominally 100 µm in width, comprised less than half of this volume (at approximately 514,290 µm ^3^).

## Data and software availability

Custom Python/PyQT control software available at DOI: 10.5281/zenodo.192280 ( Jones, 2016).

SBF-SEM jitter data available at DOI: 10.5281/zenodo.192294 ( Brama *et al.*, 2016a).

Raw image data as TIFF stacks available at DOI: 10.5281/zenodo.192470 ( Brama *et al.*, 2016b).

*Figshare:* Supplementary Movie S1. Serial sectioning in a microtome with ultraLM fluorescence imaging. doi: 10.6084/m9.figshare.4291583.v1 ( Brama *et al.*, 2016c).

*Figshare:* Supplementary Movie S2. Serial blockface fluorescence imaging in SBF-SEM with miniLM. doi: 10.6084/m9.figshare.4291586.v1 ( Brama *et al.*, 2016d).

*Figshare:* Supplementary Movie S3. Serial blockface scanning electron microscopy imaging with miniLM in-situ. doi: 10.6084/m9.figshare.4291589.v1 ( Brama *et al.*, 2016e).

## Supplementary material

Supplementary Movie S1. Serial sectioning in a microtome with ultraLM fluorescence imaging. Slices acquired at 500 nm thickness with the ultraLM mounted on an ultramicrotome. Cells below the surface initially appear as blurred objects, come into focus as they approach the cutting plane and then disappear as they are physically removed from the block by the diamond knife. Copyright: © 2016 Brama E et al.2016Data associated with the article are available under the terms of the Creative Commons Zero "No rights reserved" data waiver (CC0 1.0 Public domain dedication).

Supplementary Movie S2. Serial blockface fluorescence imaging in SBF-SEM with miniLM. Slices acquired at 100 nm thickness with the miniLM operating inside the scanning electron microscope. The outer circle shows the edge of the fibre bundle and the effect of the discrete fibre cores is seen as a fixed-pattern pixellation across the surface. Copyright: © 2016 Brama E et al.2016Data associated with the article are available under the terms of the Creative Commons Zero "No rights reserved" data waiver (CC0 1.0 Public domain dedication).

Supplementary Movie S3. Serial blockface scanning electron microscopy imaging with miniLM in-situ. Slices acquired at 100 nm thickness, miniLM images of in-situ fluorescence on the left, SBF-SEM images on the right. Copyright: © 2016 Brama E et al.2016Data associated with the article are available under the terms of the Creative Commons Zero "No rights reserved" data waiver (CC0 1.0 Public domain dedication).
