# Supplementary material for: ultraLM and miniLM: Locator tools for smart tracking of fluorescent cells in correlative light and electron microscopy
Source: Wellcome Open Res. 2016 Dec 13;1:26. [Version 1] doi: 10.12688/wellcomeopenres.10299.1 (PMC5234702; doi:10.12688/wellcomeopenres.10299.1)
Supplement: Peer review history

# Peer review: Referee response for version 1

**DOI:** 10.21956/wellcomeopenres.11092.r18611

**Published:** 2017-01-11

**Authors:** Thomas H. Sharp, Maarten Tuijtel

**Affiliations:** 1 Section Electron Microscopy, Department of Molecular Cell Biology, Leiden University Medical Center (LUMC), Leiden, Netherlands; 2 Leiden University Medical Center, Leiden, Netherlands

Brama et al. describe the development of two fluorescent microscopes to aid the experimenter in finding back cells or regions of interest in IRF blocks, while sectioning the block. One of these microscopes, the ultraLM, can be incorporated into an ultramicrotome, whilst the other, dubbed miniLM, can be built into a block-face SEM. By monitoring the fluorescent signal from an IRF block while sectioning, the researcher can target specific regions of interest which are labelled with a fluorophore, thereby making the sectioning and imaging much more efficient, and enabling future automation. Because the limited space available in these devices, integrating the fluorescent microscopes meant compromises had to be made, for instance, on the final resolution of the microscopes.

 In the manuscript, the authors very clearly explain the requirements and the construction of these devices, and show the workings of the microscopes by using GFP-H2B expressing cells as proof-of-principle samples. Furthermore, the authors anticipate that the miniLM can be used in the future to allow smart-tracking of specific regions within cells or tissue in the block-face SEM.

 Despite the limited resolution, we anticipate that both the ultraLM and the miniLM will be useful for processing IRF sample blocks containing sparse samples, or rare events, labelled with endogenously expressed fluorophores, and will fasten the imaging process significantly. However, we do have some recommendations to the authors, which are listed as follows:

1. In the design requirements for each microscope, the authors specify a minimum resolution that should be obtained, in both cases 1 µm. For both microscopes however, this requirement is not met. For the ultraLM, the theoretical resolution of the system, however, was calculated to be ~600 nm. The authors do not further address this discrepancy in the desired, the theoretical and the actual resolution, although in the discussion several improvements are suggested to improve the resolution.
2. For the resolution measurement of the miniLM, in Figure 4E, a smoothing filter was used, according the Methods section. We recommend also mentioning this in the legend of Figure 4, for clarity, and describing the exact filter used (mean, Gaussian, etc).
3. In the text, and the Methods section, it is mentioned that an *achromatic* lens is used for the ultraLM. However, Thorlabs component A240-A, as mentioned in the Methods section, is an *aspheric* lens. Either the wrong part number is being used, or the text should be adapted. Since only single colour microscopy is being done here, we do not see any problems with using an aspherical lens.
4. It would be valuable to include a mention of the sensitivity of the microscopes. For instance, how much fluorescence should be present in the sample to be detected? We believe this is useful information for other prospective users of the systems.
5. Aligning the fluorescence and EM images of Figure 7C would allow the reader to directly see the correlation between the two modalities, which is not clear to us.
6. To our understanding, Figure 2D is a top view of the miniLM. For clarity, we advise the authors to mention this in the figure legend.
7. As a general remark, the figures presenting microscope images could be made clearer to the reader. For instance, Figure 3D could depict the fluorescence and electron images separately as well as an overlay.

We have read this submission. We believe that we have an appropriate level of expertise to confirm that it is of an acceptable scientific standard.

# Peer review: Referee response for version 1

**DOI:** 10.21956/wellcomeopenres.11092.r18595

**Published:** 2017-01-05

**Authors:** Rainer Kaufmann

**Affiliations:** 1 Division of Structural Biology, Wellcome Trust Centre for Human Genetics, University of Oxford, Oxford, UK

The article by Brama et al. describes two custom built fluorescence microscopes to assist the CLEM workflow of resin embedded samples. The ultraLM can be attached to an ultramicrotome to record FM images of the resin block while cutting sections. The miniLM was designed to be integrated into an SEM for serial block face imaging. Both systems will be of great use for the CLEM community. As pointed out by the authors, often only a sparse population of the cells show the fluorescent label. Being able to identify these cells while cutting sections or to allow following particular cells in block face imaging will make the often tedious CLEM workflow substantially more efficient. The article provides a detailed description of the necessary hardware and optical components and a link to the python software for controlling the microscopes.

 The authors have characterized the resolution of both FM imaging systems and have demonstrated that fluorescent cells can be identified and correlated with the EM images. Therefore, both miniLM and ultraLM serve as excellent “fluorescence-guided cell locator tools”. Currently, it seems that the relatively limited resolution of both systems is the biggest obstacle for identifying subcellular features in FM. For both systems Brama et al. have self-imposed a resolution requirement of “at least 1 μm for subcellular feature recognition”. This resolution has not been reached and subcellular features have not been shown in the example images. In the case of the miniLM, the optical fibre seems to be the most limiting component. Future designs that provide a window might not require this. A temporary solution might be to increase the magnification of the miniature objective to decrease the effect of the individual cores in the fibre on the resolution, but at the expense of the available FOV. For the ultraLM, a Zemax (or similar software) model might help to identify what causes the decreased optical performance of the imaging lens compared to the theoretical value and how to improve the optical design.

 In Fig. 1 numbering the sections or encircling them with different colours might help to illustrate which are the same in FM and EM and which are different ones (i.e. not CLEM images of the same section). FM images of a cut section will only show signals from this section. The images from the ultraLM are taken from the whole remaining block and considering the large depth of focus, the fluorescent signals in utlraLM images are originating from a volume of >30 μm thickness. This might be important to mention also in the discussion, as the ultraLM allows to find quicker the fluorescently labelled cells as compared to FM imaging in a separate microscope, but (for samples that consist of more than a 2D layer of cells) it is less specific as the fluorescent cell can only be located with a relative low precision in the axial direction.

I have read this submission. I believe that I have an appropriate level of expertise to confirm that it is of an acceptable scientific standard.

# Peer review: Referee response for version 1

**DOI:** 10.21956/wellcomeopenres.11092.r18950

**Published:** 2017-01-05

**Authors:** Harald F. Hess

**Affiliations:** 1 Janelia Research Campus, Howard Hughes Medical Institute (HHMI), Ashburn, VA, USA

This manuscript describes 2 different “piggy back” custom fluorescent microscopes that can guide volume 3D EM imaging and give a basic level of correlative light and electron microscopy. This is largely a technical apparatus description and is well stated and easy to understand. This microscope is designed in a small form factor so as not to interfere with the normal operation of cutting. In the first case it adds fluorescent optical imaging from the exposed sample surface that is mounted on a commercial ultra microtome. An even more compact microscope is mounted on a serial block face system (3 view Gatan which generates 3D SEM images) cutting assembly and fits in the few mm between the SEM and the sample. These are clever implementations of correlative microscopy worth noting. By virtue of the forced lower numerical aperture the magnification is lower and constrained to cell identification applications. This does have the feel of a first pass at the microscope and the authors do suggest improvements for known issues (e.g. the accelerometer for position accuracy). Still some deeper carefully considered elaboration for a second generation system would be helpful. I would have one further recommendation: To really make explicit the “correlation” between light and electron images. It would be very valuable to see corresponding specific features in both of the images, but I can’t pick this out from the figures or movies. Corresponding and properly oriented and aligned cropped regions should be shown side by side and by overlay to really make this important point to the reader. This should be in the existing data and would directly illustrate the main claim of the paper for potential for cell identification in SEM and light images.

I have read this submission. I believe that I have an appropriate level of expertise to confirm that it is of an acceptable scientific standard, however I have significant reservations, as outlined above.

# Peer review: Referee response for version 1

**DOI:** 10.21956/wellcomeopenres.11092.r18432

**Published:** 2016-12-21

**Authors:** Eija Jokitalo, Ilya Belevich

**Affiliations:** 1 Electron Microscopy Unit, Institute of Biotechnology, University of Helsinki, Helsinki, Finland

The article introduces two miniature fluorescent light microscopes, ultraLM and miniLM, that can be used as locator tools for finding and following fluorescent cells in in-resin fluorescence (IRF) blocks. The ultraLM can be integrated with an ultramicrotome to detect fluorescence within the block during sectioning. Proof-of-principle ultraLM operation was demonstrated using IRF blocks containing cultured cells expressing GFP-H2B in nucleus. Although this specimen served the purpose of showing that the fluorescence imaging can be conducted during sectioning, it does not bring out the full potential of the system, as GFP-positive cells were spread evenly within the cell pellet. The ultraLM will benefit greatly applications with sparsely-labelled cell populations and genetically-modified model organisms where only subset of tissue is positive, as explained in the discussion.

 The miniLM operates inside the scanning electron microscope (SEM) chamber in conjunction with serial block face imaging (SBF-SEM). Despite the tight space constrains of the SBF-SEM the authors were able to fit a fluorescent microscope into the system. The system demonstrates decent quality of the florescent signal and has potential for navigation of SBF-SEM imaging to the areas of interest once the software for the smart-tracking is ready. The article provides electronic scheme and the control software enabling replication of the instruments elsewhere.

 It seems that the weakness of the system is related to the hardware part of the miniLM leading to rather large inter-step deviation (as shown on Figure 7 and Movie S3) that may hamper proper correlation between EM and LM images and development of more automated system in the future. The current design is using an accelerometer to identify the moment when LM image should be taken. Would it be possible that the low precision of the accelerometer is  bigger contributing factor to the inter-step deviation than the mechanical instability or jitter caused by electric noise? Have the authors considered any other alternative to detect the stopping point? For example, high precision linear or rotary optical encoders have resolution up to several nanometers.

 We have two recommendations for changing the manuscript.

1. The authors provide the control software written in the Python programming language for collecting and saving images. However, the provided functions cannot be used alone and have multiple dependencies. It would be nice if all required dependencies were additionally listed in the manuscript.
2. Figure 8 illustrates the workflow for automation of integrated 3D LM and EM using the miniLM. Images are somewhat misleading, as the size of the fluorescent spot in LM and organelle profiles in EM images are about 10 times smaller than the operation scale of the miniLM. Resolution of the miniLM system has to be improved significantly before it enables single organelle tracking. Images exemplifying cell tracking might be more appropriate.

We have read this submission. We believe that we have an appropriate level of expertise to confirm that it is of an acceptable scientific standard.
